# Machine Learning for COVID-19 Determination Using Surface-Enhanced Raman Spectroscopy

**DOI:** 10.3390/biomedicines12010167

**Published:** 2024-01-12

**Authors:** Tomasz R. Szymborski, Sylwia M. Berus, Ariadna B. Nowicka, Grzegorz Słowiński, Agnieszka Kamińska

**Affiliations:** 1 Institute of Physical Chemistry, Polish Academy of Sciences, Kasprzaka 44/52, 01-224 Warsaw, Poland; sberus@ichf.edu.pl; 2 Institute for Materials Research and Quantum Engineering, Poznan University of Technology, Piotrowo 3, 60-965 Poznan, Poland; anowicka@ichf.edu.pl; 3 Department of Software Engineering, Warsaw School of Computer Science, Lewartowskiego 17, 00-169 Warsaw, Poland; gslowinski@ms.wwsi.edu.pl

**Keywords:** surface-enhanced Raman spectroscopy, SERS, SARS-CoV-2, machine learning, random forest classifier

## Abstract

The rapid, low cost, and efficient detection of SARS-CoV-2 virus infection, especially in clinical samples, remains a major challenge. A promising solution to this problem is the combination of a spectroscopic technique: surface-enhanced Raman spectroscopy (SERS) with advanced chemometrics based on machine learning (ML) algorithms. In the present study, we conducted SERS investigations of saliva and nasopharyngeal swabs taken from a cohort of patients (saliva: 175; nasopharyngeal swabs: 114). Obtained SERS spectra were analyzed using a range of classifiers in which random forest (RF) achieved the best results, e.g., for saliva, the precision and recall equals 94.0% and 88.9%, respectively. The results demonstrate that even with a relatively small number of clinical samples, the combination of SERS and shallow machine learning can be used to identify SARS-CoV-2 virus in clinical practice.

## 1. Introduction

The rapid, accurate, and inexpensive detection of viruses in clinical samples took on new importance with the outbreak of the COVID-19 pandemic [1]. Two types of tests for SARS-CoV-2 were in widespread use: (i) molecular diagnostic test for the detection of viral genetic material (RNA) in patient’s sample and (ii) serologic test for the detection of immune response against the SARS-CoV-2 virus. The former (e.g., RT-PCR [2]) possess very high sensitivity and specificity; however, a number of factors can lead to incorrect results (e.g., virus mutation, PRC inhibition, etc.) and the technique is time consuming and requires expensive reagents [3,4]. The latter techniques (e.g., chemiluminescent methods, ELISA, antibody tests) strongly depend on the kinetics of SARS-CoV-2 antibody, and thus the optimization of the tests and interpretation of the results can be challenging. Additional detection methods for SARS-CoV-2 virus include optical biosensors [5], electrochemical [6,7,8], FET biosensor [9], colorimetric detection [10], and others [11,12,13].

In recent years, spectroscopic techniques such as surface-enhanced Raman spectroscopy (SERS) are increasingly being used for the detection of viruses using indirect and direct methods [14,15,16,17]. Indirect detection uses an SERS tag: Raman reporter molecule (e.g., p-MBA) with the recognition element (specific antibody) and capture substrate, which is functionalized with a capture antibody. The measurement is based on tracking the Raman signal from the reporter molecule, which is a more sensitive and specific method than the direct method. SERS is well known for its incredibly high sensitivity, allowing for the detection of even a single molecule. In addition, it is a fast, simple, reagent free, and non-destructive method for sample analysis. For these reasons, SERS recently gained attention as a prospective technique in biosensing [18], where SARS-CoV-2 is of special interest. Particularly promising is the combination of the highly efficient SERS technique with advanced chemometric methods, especially those based on artificial intelligence methods (e.g., machine learning or deep learning).

Machine learning was previously demonstrated for the identification of bacteria using a combination of SERS and deep learning algorithms [19,20,21], the detection of biomarkers for head and neck cancer samples [22], and the detection of Sjogren’s syndrome and diabetic nephropathy [23]. SERS and machine learning techniques were also used to differentiate between samples containing influenza A and B viruses with an accuracy of 93% at a concentration of 200 μg/mL [24]. One of the cutting-edge trends in analytics is combining SERS with advanced chemometric, especially in machine learning methods [25,26]. This approach is especially attractive for the detection and identification of the SARS-CoV-2 virus [27,28,29,30], especially in clinical samples [31]. Ikponmwoba et al. [32] used SERS and machine learning for the detection of COVID-19 in biological samples obtained from 20 patients. The authors performed dimensionality reduction using PCA and nonlinear dimensionality reduction using UMAP (Uniform Manifold Approximation and Projection). Finally, they used Gaussian process (GP) classification to predict the occurrence of a negative or positive sample as a function of low-dimensional space variables. The GP classifier provided a probability, either 0 or 1, to classify whether a sample was COVID-negative or COVID-positive. Karunakaran et al. [33] demonstrated the use of the label-free SERS technique and machine learning for the analysis of saliva samples in healthy COVID-19-infected and COVID-19-recovered patients. The proposed method could also differentiate the three classes of corona virus spike protein, i.e., SARS-CoV-2, SARS-CoV, and MERS-CoV. The authors used trained support vector machine (VSM) and achieved high accuracy predictions for healthy and COVID-19-infected patients, respectively. Yang et al. [14] developed a label-free diagnostic platform, which combines SERS and machine learning algorithms for the detection of thirteen viruses, e.g., SARS-CoV-2, SARS-CoV-2 B1, CoV 229E, IBV, HMPV-A, and others. The samples were prepared in the laboratory, where the viruses were propagated in Vero E6 cells, and after 48 h, the viruses were harvested with the procedure described in the article. The authors used support vector machine (SVM), k-nearest neighbor, and random forest algorithms. Yang et al. [34] developed a sensor with a deep learning algorithm for the detection of SARS-CoV-2 in human nasopharyngeal swabs. The authors prepared the sensor using a silver nanorod array substrate by assembling DNA probes to capture SARS-CoV-2 RNA. For chemometric analysis, the authors used a recurrent neural network (RNN)-based deep learning model. They classified 40 positive and 120 negative samples with an accuracy of 98.9%. Hwang et al. [35] reported an SERS face mask for the label-free detection of the aerosolized SARS-CoV-2 virus using Au-TiO_2_ nanocomposites. The SERS platform was placed on the inside of the face mask, where the Au-TiO_2_ SERS face mask continuously preconcentrated and efficiently captured the oronasal aerosols. An autoencoder neural network was then employed for the accurate classification of the SARS-CoV-2 virus at various concentrations. Ansah et al. [36] presented the identification of viruses via a combination of SERS with pathogen-mediated composite materials on Au nanodimple electrodes and ML. Viruses were trapped in 3D plasmonic concave spaces via electrokinetic pre-concentration, leading to ultrasensitive SERS detection. The authors used two ML models, SVM and CNN, for the specific identification of eight virus species, including influenza A viruses, human rhinovirus, and human coronavirus.

The same set of spectral data presented in this manuscript, both saliva and nasopharyngeal swabs, have been analyzed by the authors through chemometric methods using commercial software Unscrambler^®^ (CAMO software AS, version 10.3, Oslo, Norway). The results and the use of typical chemometrics in the analysis of clinical samples infected with SARS-CoV-2 virus was presented by Berus et al. [37] The analysis was divided into two steps: (i) preparation and optimization of calibration models and (ii) external validation using samples with an unknown origin (CoV+ or CoV−) for checking the classification abilities of previously created calibration models. Based on the analysis with different methods (PLS-DA, SVMC, and PCA-LDA), we indicated that the best COVID-19 diagnosis can be delivered via the SVMC method over saliva samples. Such an analysis results in impressive diagnostic parameters both on the calibration (sensitivity 100%, specificity 100%, and accuracy 100%) as well as the validation step (sensitivity 100%, specificity 80%, and accuracy 90%). The PCA-LDA and PLS-DA methods also deal well with saliva samples as the accuracy equals 80% and the sensitivity 90% or 100% (validation). We also demonstrated that the nasopharyngeal swabs can be an equally convenient methodology as SVMC presents sensitivity and accuracy at the level of 88% and 75%, respectively. The relatively low specificity of 69% can be upgraded via PLS-DA and PCA-LDA methods, which would result in 75% for both cases.

This article demonstrates the SERS technique for a fast and simple measurement of the clinical samples: saliva and nasopharyngeal swabs. In the next step, we applied machine learning algorithms, namely gaussian naive Bayes (GNB), random forest (RF), support vector classifier (SVC), and logistic regression (LR). We tested them regarding classification abilities expressed by diagnostic parameters (accuracy, precision, and recall). The results show that ML, even for sets of data considered as small, can classify samples with reasonable precision and accuracy. Lastly, we compared the results with the analysis performed using typical chemometric methods. This study is an extension of the previous one by Berus et al. [37], where chemometric methods (via Unscrambler software, CAMO software AS, version 10.3, Oslo, Norway) were tested on the same spectral data. With support machine classification, we reached the sensitivity and accuracy of 100% and 90%. This research is also an ideal complement as the RF algorithm can improve the precision to 90%.

## 2. Materials and Methods

### 2.1. Clinical Samples

Clinical samples from 289 patients of saliva and nasopharyngeal swabs were collected from the Department of Clinical Genetics, Medical University of Łódź (Łódź, Poland) and stored in frozen form at −80 °C. The samples, prior to storage at low temperature, were tested for SARS-CoV-2 via the qRT-PCR method, according to the guidelines of the Department of Clinical Genetics, Medical University of Łódź (Łódź, Poland). The extraction of viral RNA of SARS-CoV-2 was performed with the use of chemagic 360 automated extraction platform (PerkinElmer, Naperville, IL, USA). To verify the presence of SARS-CoV-2 in the samples, the following methods were used: qRT-PCR amplification of open reading frame 1ab (ORF1ab), nucleocapsid protein (NP) gene fragments, and positive reference gene using DiaPlexQ Novel Coronavirus (2019-nCoV) Detection Kit (SolGent Co, Ltd., Daejeon, Republic of Korea). Detailed information on the extraction of RNA and qRT-PCR measurements can be found in our previous article by Berus et al. [37].

### 2.2. Measurements of the Clinical Samples

SERS measurements require using cost-effective SERS platforms in large quantities, which provide high enhancement factor (EF), reproducibility, and stability over time. For this purpose, we have used SERS platforms based on femtosecond laser-modified silicon [38]. The silicon was modified using a femtosecond laser (λ = 1030 nm) with a repetition rate of 300 kHz and a pulse of 300 femtoseconds. The modification occurred on mechanically pre-cut silicon squares (3 mm × 3 mm), thus obtaining a reproducible and large number of SERS platforms for later use. The final step of the procedure was the sputtering of 100 nm of silver using the PVD device (Quorum, Q150T ES, Laughton, UK) using 25 mA current. SERS-active platforms were stored in an inert gas atmosphere.

The samples were conditioned at room temperature, and then ca. 2 μL of liquid from each sample was pipetted onto the SERS-active platform. The platform with the liquid sample was attached to a glass slide and placed under a laminar flow cabinet, typically for 2–3 min, to evaporate the water. Then, the glass slide was placed under a spectrometer so the laser beam was in the very center of the SERS platform. The measurements were performed using a BRAVO (Bruker, Rosenheim, Germany) spectrometer equipped with a Duo Laser system (700–1100 nm, 100 mW) and a CCD camera. The spectral resolution was 2–4 cm^−1^. Typically, 30 SERS spectra were recorded for a single sample, and the time of acquisition for a single spectrum was 30 s.

**Figure 1:**
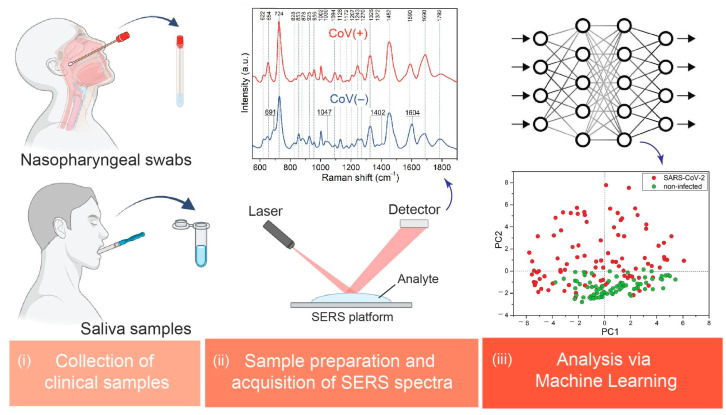
Procedure consists of three main steps: (i) collection of clinical sample, (ii) sample preparation and spectra acquisition, and (iii) machine learning analysis. Figure created with BioRender.com.

SERS spectra were pre-processed using OPUS software (Bruker Optic GmbH, ver. 2012). The raw spectra were subjected to smoothing using a Savitzky–Golay filter (five points), baseline concave rubber band correction with six iterations (five baseline points), cutting to the range between 600 cm^−1^ and 1700 cm^−1^, and finally, Min–Max normalization. Such pre-processed spectra were subjected to machine learning analysis. The whole procedure of measurement and analysis is presented in Figure 1.

### 2.3. Machine Learning Analysis

#### 2.3.1. Principal Component Analysis (PCA)

In the Principal Component Analysis (PCA) method, the correlated data are reduced into uncorrelated data presented in a dimension described by so-called principal components (PCs). The method is based on bilinear decomposition mathematically described as:

X = TP^T^ + E

where:

X—Initial matrix of data;
T—Scores matrix;
P—Loading matrix;
E—Error matrix.

PC scores are related to the linear combination of the original variables and describe the differences and similarities between samples. The first principal component (PC-1) accounts for the most significant variance in the data. The loadings describe the data structure concerning the correlation of the variables and show how well a PC takes the variation of these variables into account. By analyzing the plot of PC loadings as a function of variables (i.e., Raman shifts), one can indicate the main diagnostic variables or regions related to the differences in the dataset [39].

#### 2.3.2. Machine Learning Classification

Machine learning (ML) classification experiments were performed with selected ML methods: gaussian naive Bayes (GNB) classifier [40], random forest (RF) [41], support vector classifier (SVC) [42], and logistic regression (LR) [43]. Default parameters were used for all classifiers. The only exception was performed for the logistic regression classifier that needed more than 100 default iterations to converge, and the number of maximal iterations was increased to 500. Data were analyzed via shallow learning techniques using Python and its frameworks for data manipulation, visualization and machine learning: numpy, pandas, matplotlib, seaborn, and scikit-learn.

## 3. Results

### 3.1. SERS Measurements and Band Assignments

In the current study, we examined clinical samples taken from 289 patients, including 175 samples of saliva and 114 samples of nasopharyngeal swabs. All patients were diagnosed with the PCR method and, depending on the result, divided into COVID-19(+) (infected with SARS-CoV-2 virus) and COVID-19(−) (non-infected with SARS-CoV-2 virus) samples. For clarity, we have labeled these samples as CoV(+) and CoV(−), respectively. The summary of the used samples is demonstrated in Table 1.

**Figure 2:**
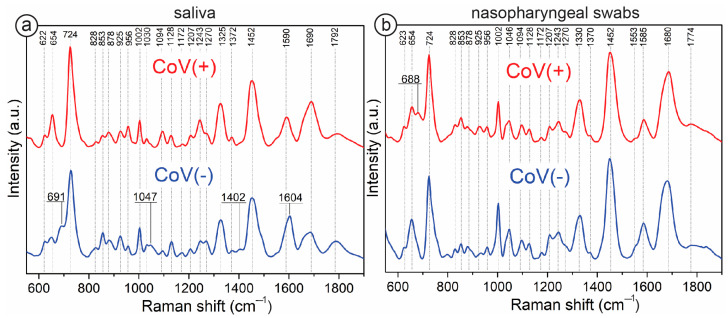
SERS spectra of COVID-19 infected (CoV(+)) and non-infected samples (CoV(−)) from saliva (**a**) and nasopharyngeal swabs (**b**).

Figure 2 demonstrates averaged SERS spectra of saliva (a) and nasopharyngeal swabs (b). The figure contains a spectrum of infected samples labeled as CoV(+) and non-infected samples labeled as CoV(−). Dashed lines mark assigned bands, whereas the continuous lines with the band value show the bands that are present in the sample (e.g., CoV(−)) and simultaneously do not exist in the other sample type, i.e., CoV(+). When comparing the averaged spectra of samples infected with SARS-CoV-2 and non-infected samples (CoV−), both saliva and nasopharyngeal swabs, we observe apparent differences between them. For CoV(−) samples of saliva (Figure 2a: CoV(−)), the most characteristic bands are at 691, 724, 853, 878, 1002, 1047, 1128, 1270, 1325, 1452, 1590, 1690, and 1792 cm^−1^. All bands were identified and their origin was described in Table S1 (see Supplementary Materials). Here, we present the origins of the most intense bands:

(i) An origin of 724 cm^−1^ corresponds to O–O stretching vibration in oxygenated proteins, glycoproteins (e.g., mucin), and to the ring breathing mode of tryptophan.
(ii) An origin of 1325 cm^−1^ corresponds to amide III band in proteins and DNA.
(iii) An origin of 1452 cm^−1^ corresponds to C–H stretching of glycoproteins, including mucin.
(iv) An origin of 1585 cm^−1^ corresponds to ring and C=C vibrations in tyrosine and phenylalanine.

Spectral changes between CoV(+) and CoV(−) reflect the differences in biochemical composition and provide information between components in saliva (e.g., the intensity ratio of 853/828 cm^−1^ can be explained by the interaction between tyrosine residues with viral proteins, other expressed molecules, and immunity proteins) [44,45]. The SERS fingerprint of saliva infected with the SARS-CoV-2 virus is characterized by bands at 654, 720, 1320, and 1443 cm^−1^, which can be assigned to specific oscillations in methionine and methionine adenosyl transferase [46]. We observed an increased intensity of these four bands for CoV(+) saliva. In relation to the band 1002 cm^−1^ with fixed intensities, the ratios are as follow: CoV(+) I_654_/I_1002_ = 1.3; I_720_/I_1002_ = 4.17; I_1320_/I_1002_ = 1.72; I_1445_/I_1002_ = 2.72 and CoV(−) I_654_/I_1002_ = 0.61; I_720_/I_1002_ = 3.3; I_1320_/I_1002_ = 1.32; I_1445_/I_1002_ = 2.25. This can be explained by the increased requirement for methionine during infection [47]. Also, recent studies demonstrated that the level of ferritin in saliva can rise during infection [48]. These increased levels of ferritin and specific immunoglobulins in saliva lead to intensified bands in region 1200–1300 cm^−1^, as well as bands at 1325, 1450, and 1690 cm^−1^ from amide III and amide I of proteins. Some band assignments can have multiple origins. Bands at 1094, 1242, and 1325 cm^−1^ originate from the phosphodiester group and purine bases of nucleic acids. Their increased intensity in CoV(+) samples of saliva can be explained by the multiplication of genetic material during infection.

The analysis of nasopharyngeal swabs demonstrated that in CoV(+) and CoV(−) samples, the strong SERS bands are located at 724, 1002, 1045, 1330, 1452, 1590, and 1680 cm^−1^. A distinct difference is observed between 680 cm^−1^ and 950 cm^−1^. For CoV(+) spectra, a new band appears at 688 cm^−1^, which is characteristic of neopterin. An increase in the 925 cm^−1^ band, assigned to carboxylates and proline rings compounds, is also observed [49,50]. The relative spectral intensity of the bands 745 cm^−1^ to 1455 cm^−1^ demonstrates efficacy for the classification of the samples. CoV(+) samples demonstrate an intensity ratio of I_724_/I_1455_ at the level 1.03 ± 0.05, whereas for CoV(−), this ratio is 1.23 ± 0.04.

The above analysis demonstrates that the analyzed biological samples are characterized by biochemical complexity and variability from patient to patient. SERS spectra, complex to analyze empirically, are excellent research material for chemometric analyses, especially those based on machine learning and artificial intelligence.

### 3.2. Principal Component Analysis (PCA)

**Figure 3:**
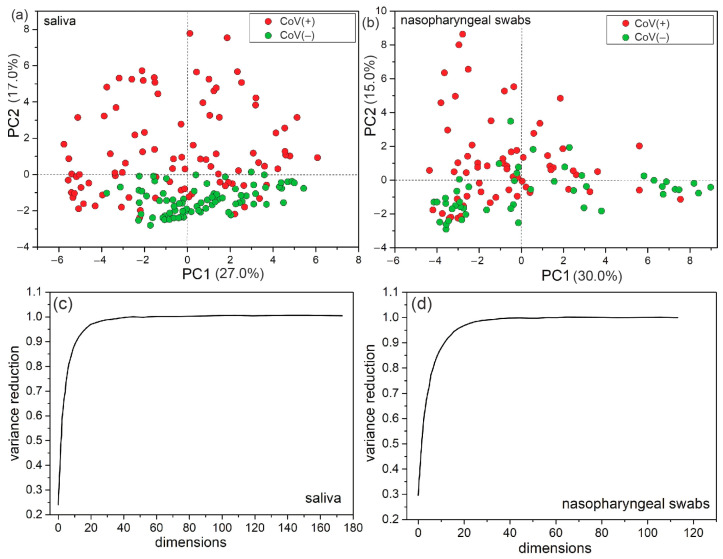
Visualization of saliva (**a**) and nasopharyngeal swab (**b**) datasets after PCA transformation to 2D space. Red dots stated as samples infected with the SARS-CoV-2 virus (CoV(+)), and green dots represent non-infected samples (CoV(−)). (**c**) Visualization of saliva and (**d**) nasopharyngeal swab variance reduction as a function of dimensions. Variance in reduction increases linearly with an increase in dimensions and achieved a plateau at dimensions of ca. 25–30.

In the first step, Principal Component Analysis (PCA) was used to reduce the dimensionality of many uncorrelated spectral data into correlated ones. In a new dimension described by principal components (PC-1, PC-2), every single spectrum is represented by a single point, and the dependencies between them are more visible. In a plot score (Figure 3), we observe that saliva samples are characterized by a more symmetrical distribution than nasopharyngeal swabs. Moreover, the spectral information explained by PC-1 and PC-2 is higher and equals 44%, while for nasopharyngeal swabs, it is 45%. Thus, we can conclude that PCA works better for saliva samples regarding CoV(+) and CoV(−) differentiation.

Dataset dimensionality can be reduced from over 500 to a smaller number with insignificant variance reduction (see Figure 3c,d). For both types of samples, the dimensionality of 20 has been chosen as the optimal value, providing the variance perseverance at the level of 96.3% and 96.3% for saliva and nasopharyngeal swabs, respectively. We considered a value of 20 to be optimal as further size increases do not lead to a significant improvement in variance reduction. A change from 20 to 40, i.e., 100%, results in an improvement of only 3%. The variance perseverance ratio as a function of a number of dimensions for both saliva and nasopharyngeal swabs are demonstrated in Table 2.

### 3.3. Classification of the Samples Using Machine Learning Algorithms

The second step in the classification of the clinical samples was the testing of selected methods of ML algorithms. For preliminary assessment, we selected four methods: Gaussian naive Bayes (GNB), random forest (RF), support vector classifier (SVC), and logistic regression (LR).

**Figure 4:**
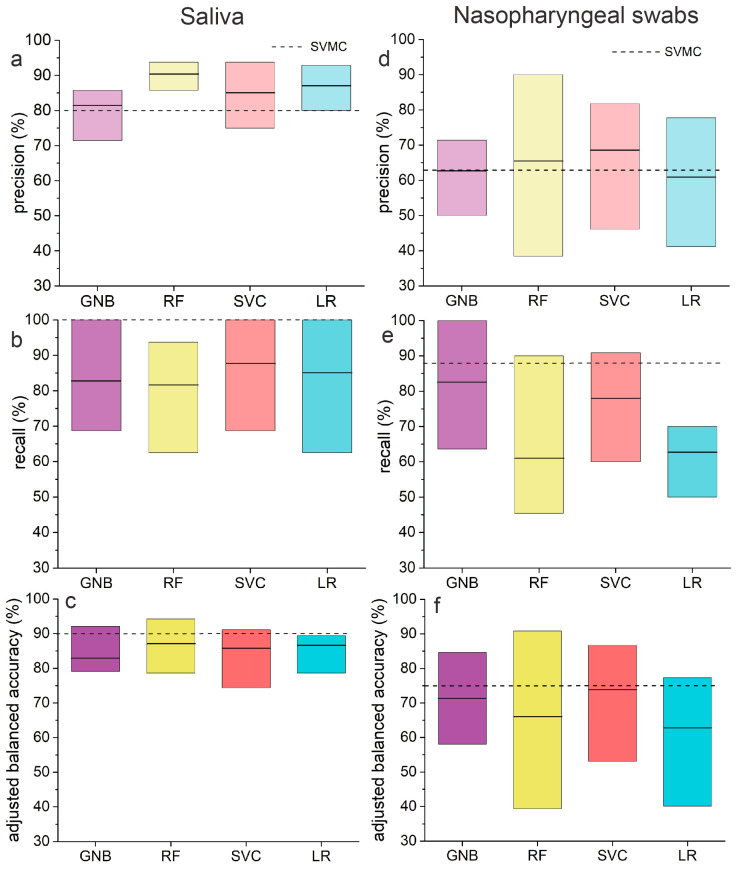
Precision (**a**,**d**), recall (**b**,**e**) and adjusted balanced accuracy (**c**,**f**) for the saliva and nasopharyngeal swabs samples. The figure presents: gaussian naive Bayes (GNB), random forest (RF), support vector classifier (SVC) and logistic regression (LR). This dataset was previously analyzed using standard chemometric methods [37], where SVMC gave the best results. The mean value obtained with SVMC is presented as a dashed line.

Single ML experiment results can be influenced by dataset random division to train and test subsets. To mitigate this effect, five-fold cross-validation has been applied with a train/test division proportion equal to 80/20. As performance metrics, we used precision, recall, and balanced accuracy. The datasets are slightly imbalanced; positive and negative class sizes are not equal, and for that reason, balanced accuracy is a better metric in such cases than ordinary accuracy. The precision, recall, and adjusted balanced accuracy values of all tested classifiers are displayed in Table 3, whereas Figure 4 presents their graphical representation as bar graphs. Figure 4 also consists of a dashed line representing the mean value for the SVMC method, which was the best chemometric method of analysis in our previous article [37].

In the case of saliva, the highest averaged parameters of precision and adjusted balanced accuracy are obtained for the RF algorithm. The value of the recall parameter reaches 81.6%, similar to GNB (82.9%). LR algorithm provides high averaged values for all parameters, precision 87.1%, recall 85.1%, and adjusted balance accuracy 86.7%, which are very similar to the one obtained from the SCV. GNB algorithm works least effectively among all considered methods as it provides the lowest averaged values of precision and adjusts balanced accuracy that equals 81.4% and 83.0%, respectively.

Considering the range of values that all tested algorithms can reach, we conclude that RF offers the highest maximal value of precision with the smallest spread of values (85.7–93.7%) and a maximum balanced accuracy of 94.2%. Although the maximum recall is 93.7%, which is lower than other methods, RF performs within 78.6–94.2% of adjusted balanced accuracy with an average value of 87.1%, which is the best result.

For nasopharyngeal swabs, SVC can identify the highest CoV(−) cases and ensure proper diagnosis as the averaged values of precisions and accuracy equals 69.0% and 74.0%, respectively. In turn, GNB can identify CoV(+) cases with the recall of 82.7%, and this is the best working algorithm for the determination of the presence of SARS-CoV-2 in nasopharyngeal swabs. The accuracy is also relatively high (71.6%). With LR and FR, the averaged values of all parameters are in the range of 61.0% and 66.0%.

For random forest, we noted the highest spread of the values of all parameters in the range between 40.0% and 90.0%. RF is the only algorithm capable of reaching the maximal values of precision and adjusted balance. GNB is characterized by the highest values of recall 63.6–100.0%, whereas LR has the lowest range: 50.0–70.0%.

To compare the results obtained using Unscrambler software (Berus et al. [37]) with the results presented here, we superimposed the data of the best-performing method—SVMC (support vector machine classification) in Figure 4 and marked them with a dashed line. The values of precision, recall, and accuracy were calculated using previously created and optimized calibration models for saliva and nasopharyngeal swabs. The number of external samples used for testing the predictive abilities of models was 20 and 16 for saliva and nasopharyngeal swabs, respectively. Considering the averaged values, all algorithms operated via machine learning can upgrade the precision provided by the SVMC technique. It is advantageous while analyzing saliva samples with the RF method (precision 90.4%). For nasopharyngeal swabs, these differences are not so substantial as all values oscillate in the range between 61.0% and 68.0%. The recall offered by SVMC is 100.0% and 88.0% and is higher than all tested methods. The values of adjusted balanced accuracy offered by all methods (machine learning; SVMC) are comparable but still higher for SVMC.

All tested algorithms perform better in recognizing and identifying the SARS-CoV-2 virus in saliva samples than in nasopharyngeal swabs, as the precision, recall, and accuracy values are higher. This makes saliva a more reliable material for COVID-19 diagnosis. Since it is minimally invasive and does not cause damage during intake, this method of analysis would be preferred.

### 3.4. Classification of Saliva Samples via Random Forest

According to cross-validation results (see Section 3.3), where we tested different methods, the best classification results were achieved for saliva samples and random forest classifier. Herein, we present detailed information about using saliva samples and RFC to detect SARS-CoV-2 virus efficiently. When analyzing these samples, we used the classic approach of dividing the samples into training and testing sections.

**Figure 5:**
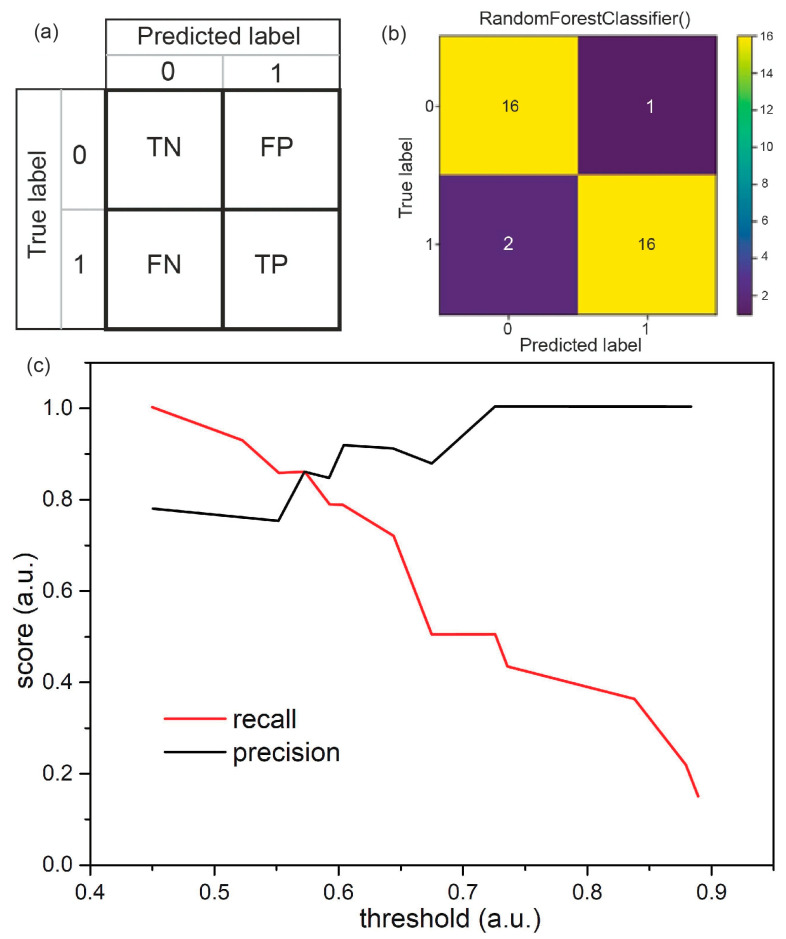
Confusion matrix scheme (**a**) and confusion matrix for RFC training process (**b**). Precision and recall as a function of the threshold for classifying a sample into class 1 (infected of the SARS-CoV-2) (**c**).

The single training results and performance achieved depend to some extent on the random samples split to train or test sets. The experience shows that this effect is more substantial with smaller sets. Figure 5a,b show a confusion matrix for single training. The full dataset contained 175 samples, and 35 samples (17 from healthy patients and 18 infected with SARS-CoV-2 virus) were put into the test set. Diagonal fields in the matrix show correctly classified samples: true negatives (TNs) and true positives (TPs). There could also be two types of mistakes. Positive samples (infected with SARS-CoV-2) could, by mistake, be classified as healthy, which are called false negatives (FNs), and truly negative samples could be recognized as positive—and are thus considered false positives (FPs).

Precision and recall were calculated according to the following equations:

$$Precision=\frac{TP}{TP+FP}$$

$$Recall=\frac{TP}{TP+FN}$$

where TP is true positive; FN is false negative; and FP is false positive.

The F-score, which is the harmonic mean of the precision and recall, can be calculated as follows:

$$F=2\times \frac{precision\times recall}{precision+recall}$$

In the case of results presented in Figure 4, the precision is 94.1%, the recall (sensitivity) is equal to 88.9 %, and the F-score is 0.914 (i.e., 91.4%). Random forest classifier (as well as other classification models) generates the probability of a given sample belonging to each class for each sample (these probabilities sum to 1). By default, a sample is classified into the class for which the probability is higher. There may be a situation when we care about greater precision or greater recall. In such a case, the following should be assessed: the costs for a false positive sample (claiming that a healthy person is infected with the virus) as well as for a false negative sample (considering the infected person as healthy). And then, the probability level obtained from a learned model should be selected, by which we classify the samples in such a way that the overall cost of mistakes was as low as possible. Figure 5c demonstrates how recall and precision change depending on the threshold value for classifying a sample into class 1 (SARS-CoV-2 infected). We also compared the results we obtained for random forest with other ML algorithms (see Table S2 in Supplementary Materials).

## 4. Conclusions

In the present work, we used clinical samples of saliva and nasopharyngeal swabs from healthy and SARS-CoV-2-infected patients to demonstrate SERS techniques and machine learning algorithms as fast and efficient methods of detecting the SARS-CoV-2 virus. Several types of shallow machine learning were tested: gaussian naive Bayes, random forest, support vector, and logistic regression. Finally, we used a random forest classifier for the analysis, and the best results were obtained for RFC and the saliva samples (averaged precision of 90.4%, averaged recall of 81.6%, and averaged adjusted balanced accuracy of 87.1%). The data were compared with the SVMC method, which was recognized in our previous work as the best chemometric method. In the case of saliva samples, RF showed greater precision and comparable adjusted balanced accuracy compared to the SVMC method, while also showing a lower recall.

The results demonstrate that ML, even for sets of data considered as small, can classify samples with reasonable precision and accuracy. The precision for random forest was 94.1%, whereas recall (sensitivity) was 88.9%, which demonstrates the potential for the practical use of combined SERS and ML methods for the detection of the SARS-CoV-2 virus in clinical samples. Hence, the RF algorithm perfectly complements the SVMC method (analyzed in our previous work), especially in terms of precision, which can be raised up to 93.7%, making this approach more accurate for diagnostic purposes. For this reason, in the future, we can create and test the multi-stage analysis of spectral data involving several methods that complement each other, i.e., SVMC and RF.

One of the most important challenges in diagnosis is detecting infected individuals at an early stage of infection. Such a task is essential for easily transmissible viruses such as SARS-CoV-2. Thus, one of the future directions for the use of SERS and machine learning would be to identify specific features of SERS spectra of saliva or nasopharyngeal swabs at an early stage of infection.

## Figures and Tables

**Table 1:** Sets of saliva and nasopharyngeal samples.

Type	Total Number of Samples	CoV(+)	CoV(−)
saliva	175	81	94
nasopharyngeal swab	114	51	63
total:	289	132	157

**Table 2:** Variance perseverance ratio for selected dimensions during PCA transformation for saliva and nasopharyngeal swabs. For both types of samples, the optimal number of dimensions was set to 20.

Number of Dimensions	Variance Perseverance Ratio (%)	
	Saliva	Nasopharyngeal Swab
1	24.5	30.0
2	41.3	44.5
5	70.0	70.0
10	86.8	86.3
20	96.3	96.3
30	98.6	98.8
40	99.4	99.5

**Table 3:** Results of cross-validation of different ML techniques for saliva and nasopharyngeal swabs. Minimal, maximal, and average (bold) results are presented.

Classifier Type	Saliva			Nasopharyngeal Swabs		
	Precision (%)	Recall (%)	Adjusted * Balanced Accuracy (%)	Precision (%)	Recall (%)	Adjusted ** Balanced Accuracy (%)
gaussian naive Bayes (GNB)	71.4	68.7	79.1	50	63.6	58.1
	85.7	100	92.1	71.4	100	84.6
	**81.4**	**83**	**83**	**62.6**	**82.7**	**71.5**
random forest (RF)	85.7	62.5	78.6	38.4	45.4	39.4
	93.7	93.7	94.2	90	90	90.8
	**90.4**	**81.6**	**87.1**	**65.7**	**61.1**	**66.1**
support vector classifier (SVC)	75	68.7	74.3	46.1	60	53.1
	93.7	100	91.1	81.8	90.9	86.7
	**85**	**87.7**	**85.8**	**69**	**78.2**	**74**
logistic regression (LR)	80	62.5	78.6	41.2	50	40.1
	92.8	100	89.5	77.8	70	77.3
	**87.1**	**85.1**	**86.7**	**61.2**	**62.7**	**62.8**

* The result is adjusted for chance, so that random performance would score 0, while keeping perfect performance at a score of 1. ** The result is adjusted for chance, so that random performance would score 0%, while keeping perfect performance at a score of 100%.

## Data Availability

Dataset available on request from the authors.

## Supplementary Materials

The following supporting information can be downloaded at: https://www.mdpi.com/article/10.3390/biomedicines12010167/s1, Figure S1: Surface of the SERS platform (silicon micromachined via femtosecond laser and covered with 100 nm of silver) acquired by Scanning Electron Microscopy (SEM); Table S1: Tentative assignments for the main bands observed in the SERS spectra of SARS-CoV-2-infected CoV(+) and healthy CoV(−) subjects [51,52,53,54,55,56,57,58]; Table S2: The comparison of accuracy, sensitivity, and specificity for the analysis of body fluids in terms of COVID-19 diagnosis in a label-free manner [33,34,59,60,61].
