# Supplementary material for: Machine Learning for COVID-19 Determination Using Surface-Enhanced Raman Spectroscopy
Source: Biomedicines. 2024 Jan 12;12(1):167. doi: 10.3390/biomedicines12010167 (PMC10813688; doi:10.3390/biomedicines12010167)
Supplement: Supplementary file 1 [file biomedicines-12-00167-s001.zip › biomedicines-2761428-supplementary.pdf]

Electronic Supporting Information

Machine Learning for COVID-19 determination using Surface Enhanced Raman Spectroscopy

Tomasz Szymborski[#1]*, Sylwia M. Berus[#1], Ariadna Nowicka[3], Grzegorz Słowiński[2] and Agnieszka Kamińska[1]*

*corresponding author: tszymborski@ichf.edu.pl, akaminska@ichf.edu.pl

# equal contribution

[1]Institute of Physical Chemistry, Polish Academy of Sciences, Kasprzaka 44/52, 01-224 Warsaw, Poland
[2]Warsaw School of Computer Science, Lewartowskiego 17, 00-169 Warsaw, Poland
[3]Institute for Materials Research and Quantum Engineering, Poznan University of Technology, Piotrowo 3, 60-965 Poznan, Poland

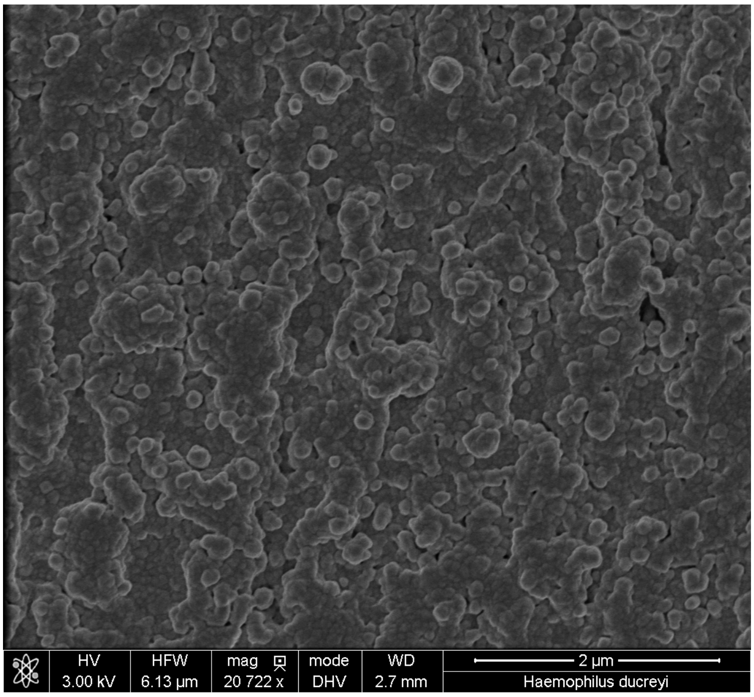

Figure S1. Surface of the SERS platform (silicon micromachinned via femtosecond laser and covered with 100 nm of silver) acquired by Scanning Electron Microscopy (SEM).

Table S1. Tentative assignments for the main bands observed in the SERS spectra of SARS-CoV-2 infected CoV(+) and healthy CoV(-) subjects.

**Sample**				**Assignment**
**Saliva**		**Nasopharyngeal swabs**		
**CoV(-)**	**CoV(+)**	**CoV(-)**	**CoV(+)**	
622	622	623	623	adenine, C-C twisting mode of phenylalanine (protein)
649	654	654	654	C-S stretching vibration in methionine<br>C-C twisting mode of tyrosine
			679	Ring breathing modes in the DNA bases,<br>G (ring breathing modes in the DNA bases) neopterin
691				δ(O–C=O) Creatinine, cytosine
724	724	724	724	O-O stretching vibration in oxygenated proteins, glycoproteins like mucines, ring breathing mode of tryptophan (protein assignment), C-N head group choline $(H_3C)_3N^+$ (lipid assignment)
828	828	828	828	Ring breathing mode of tyrosine, Transferrin (Tyrosine, H-bonding)
853	853	853	853	Ring breathing mode of tyrosine, Transferrin (Tyrosine, H-bonding)
878	878	878	878	Proline, valine, glycine, tryptophan, glutamate or ν (C─C) Hydro-oxyproline, Transferrin (Tryptophan, H-bonding) or $\nu_s P(OH)_2$ of phosphate
925	925	925	925	C-C stretching proline ring, carboxylates including glucose and glycogen
956	956	956	956	hydroxyapatite, xanthine<br>proline, valine
1002	1002	1002	1002	aromatic ring breathing of phenylalanine<br>phenylalanine in Lysozyme, lactoferrin, albumin,<br>Transferrin (Phenylalanine)
1030	1030			C-H in-plane bending mode of phenylalanine<br>Phenylalanine in Lysozyme, lactoferrin, albumin
1047		1046	1046	C-O and C-N stretching in proteins, Glycogen $C–CH_3$ vibration
1094	1094	1094	1094	Symmetric PO_2^- stretching vibration of the DNA backbone<br>T cells
1128	1128	1128	1128	C-O stretching (carbohydrates), C-N stretching (proteins)
1172	1172	1172	1172	bending C-H tyrosine, Transferrin (Tyrosine, CH3)
1207	1207	1207	1207	tryptophan and phenylalanine $\nu(C\text{-}C_6H_5)$ mode, Hydroxyproline, tyrosine<br>Tryptophan in Lysozyme, lactoferrin, albumin
1243	1243	1243	1243	phosphodiester group associate with nucleic acid

				B-sheet (the most common secondary structures in proteins e.g. alfa amylase)
1270	1270	1270	1270	Stretching C-N, bending N-H - amide III band in proteins<br>Transferrin (Tyrosine/α-helix)<br>α-helix (the most common secondary structures in proteins e.g. alfa amylase)
1325	1325	1325	1325	amide III band in proteins<br>CH_3CH_2 wagging mode in purine bases of nucleic acids<br>T cells
1372	1372	1372	1372	Lipids, proteins (tryptophan)<br>T, A, G (ring breathing modes of the DNA/RNA bases)<br>T cells
1402				Bending of methyl groups in proteins
1452	1452	1452	1452	the C-H stretching of glycoproteins including mucines or Hydrocarbon chain of lipid, Triglycerides CH_3 Deformation of lipids<br>CH2, CH3 bend of tryptophan<br>Tryptophan in Lysozyme, lactoferrin, albumin<br>T cells
Shoulder<br>1550	Shoulder<br>1550	Shoulder<br>1553	Shoulder<br>1553	υ(CN) and δ(NH) amide II<br>ν (C=C) tryptophan
1604	1590	1585	1585	phenylalanine, tryptophan, hydroxyproline, hypoxanthine<br>C=C in-plane bending mode of phenylalanine & tyrosine<br>Cytosine (NH_2)
1690	1690	1680	1680	Amide I of proteins (Lysozyme, lactoferrin, albumin)

Literature:

Lin, X.; Lin, D.; Ge, X.; Qiu, S.; Feng, S.; Chen, R. Noninvasive Detection of Nasopharyngeal Carcinoma Based on Saliva Proteins Using Surface-Enhanced Raman Spectroscopy. J. Biomed. Opt. 2017, 22, 105004, doi:10.1117/1.JBO.22.10.105004.

Li, X.; Yang, T.; Lin, J. Spectral Analysis of Human Saliva for Detection of Lung Cancer Using Surface-Enhanced Raman Spectroscopy. J. Biomed. Opt. 2012, 17, 037003, doi:10.1117/1.JBO.17.3.037003.

Talari, A.C.S.; Movasaghi, Z.; Rehman, S.; Rehman, I.U. Raman Spectroscopy of Biological Tissues. Appl. Spectrosc. Rev. 2015, 50, 46–111, doi:10.1080/05704928.2014.923902.

Austin, L.A.; Osseiran, S.; Evans, C.L. Raman Technologies in Cancer Diagnostics. Analyst 2016, 141, 476–503, doi:10.1039/C5AN01786F.

Cao, G.; Chen, M.; Chen, Y.; Huang, Z.; Lin, J.; Lin, J.; Xu, Z.; Wu, S.; Huang, W.; Weng, G.; et al. A Potential Method for Non-Invasive Acute Myocardial Infarction Detection Based on Saliva Raman Spectroscopy and Multivariate Analysis. Laser Phys. Lett. 2015, 12, 125702, doi:10.1088/1612-2011/12/12/125702.

Oliveira, E.M.; Rogero, M.; Ferreira, E.C.; Gomes Neto, J.A. Simultaneous Determination of Phosphite and Phosphate in Fertilizers by Raman Spectroscopy. Spectrochim. Acta Part A Mol. Biomol. Spectrosc. 2021, 246, 119025, doi:10.1016/J.SAA.2020.119025.

Hu, P.; Zheng, X.S.; Zong, C.; Li, M.H.; Zhang, L.Y.; Li, W.; Ren, B. Drop-Coating Deposition and Surface-Enhanced Raman Spectroscopies (DCDRS and SERS) Provide Complementary Information of Whole Human Tears. J. Raman Spectrosc. 2014, 45, 565–573, doi:10.1002/JRS.4499.

Virkler, K.; Lednev, I.K. Forensic Body Fluid Identification: The Raman Spectroscopic Signature of Saliva. Analyst 2010, 135, 512–517, doi:10.1039/B919393F.

Table S2. The comparison of accuracy, sensitivity and specificity for the analysis of body fluids in terms of COVID-19 diagnosis in label-free manner.

**Algorithm**	**Type of sample**	**Accuracy**	**Sensitivity**	**Specificity**	**Literature**
SVM classifier	saliva	95.38%	-	-	[1]
Convolutional Neural Networks (CNNs)	saliva	89.6%	83.0%	93.0%	[2]
MLPClassifier	Nasopharyngeal swabs	85.0%	90.4%	79.1%	[3]
Logistic Regression classifier	Tracheal samples	97.2%	97.2%	96.4%	
PLS-DA	Nasopharyngeal swabs	91.0%	80.0%	100.0%	[4]
SVM	Nasopharyngeal swabs	86.0±4.0%	-	-	[5]
RandomForest	Nasopharyngeal swabs	84.0±6.0%	-	-	[5]
BP	Nasopharyngeal swabs	90.0±5.0%	-	-	[5]
CNN	Nasopharyngeal swabs	94.0±2.0%	-	-	[5]
Recurrent Neural Network (RNN)	Nasopharyngeal swabs	98.9±0.3%	-	-	[5]
RandomForest	saliva	91.4%	88.9%	94.1%	Current manuscript

[1] V. Karunakarn et al. “A non-invasive ultrasensitive diagnostic approach for COVID-19 infection using salivary label-free SERS fingerprinting and artificial intelligence”, J Photochem Photobiol B. 2022 Sep:234:112545. doi: 10.1016/j.jphotobiol.2022.112545.

[2] C. Carlomagno et al., COVID-19 salivary Raman fingerprint: innovative approach for the detection of current and past SARS-CoV-2 infections, Sci Rep 11, 4943 (2021). https://doi.org/10.1038/s41598-021-84565-3

[3] D. M Ceccon et al., New, fast, and precise method of COVID-19 detection in nasopharyngeal and tracheal aspirate samples combining optical spectroscopy and machine learning, Braz J Microbiol. 2023 Jun;54(2):769-777. doi: 10.1007/s42770-023-00923-5.

[4] A. C. C. Goulart et al., Diagnosing COVID-19 in nasopharyngeal secretion through Raman spectroscopy: a feasibility study, Lasers Med Sci. 2023 Sep 12;38(1):210. doi: 10.1007/s10103-023-03871-6.

[5] Y. Yang et al., Rapid Detection of SARS-CoV-2 RNA in Human Nasopharyngeal Specimens Using Surface-Enhanced Raman Spectroscopy and Deep Learning Algorithms, ACS Sens. 2023, 8, 1, 297–307
